# The Role of Emotional Competencies and Mentalization in Burnout Development Among Psychiatry Residents: Longitudinal Findings from the QASP Project

**DOI:** 10.1192/j.eurpsy.2025.920

**Published:** 2025-08-26

**Authors:** L. Tarchi, G. Castellini, V. Ricca, A. Fiorillo

**Affiliations:** 1 Health Sciences, University of Florence, Florence; 2 Mental Health, University of Naples, Luigi Vanvitelli, Naples, Italy

## Abstract

**Introduction:**

Burnout is a pervasive issue among psychiatry residents, with long-term consequences for both individual well-being and the quality of care provided to patients. The role of emotional competencies, including emotional regulation, self-awareness, and mentalization, is increasingly recognized as critical in mediating burnout outcomes, according to the job demands-resources model of burnout.

**Objectives:**

The study objective was to investigate predictors of burnout and burnout correlates in a sample of psychiatry trainees.

**Methods:**

This study examines longitudinal data from the QASP project (*Questionnaire and Assessment of Stress and Performance in psychiatry residents*), which also aims to assess the relationship between emotional exhaustion, depersonalization, and personal accomplishment in medical residents across multiple centers in Italy. Using a mixed longitudinal model, we explored predictors of burnout and its correlates, in particular mentalization deficits, emotional dysregulation, and attachment insecurity.

**Results:**

The study involved 827 psychiatry residents enrolled across different psychiatry training programs in Italy, enrolled in two waves: 2022 and 2023. To date, 351 follow-up assessments were retrieved. Emotional exhaustion showed a rapid increase during the early years of residency, then stabilizing in later years (Figure 1).

The results also indicated a strong relationship between increased exposure to violence and elevated burnout dimensions. However, coping strategies were observed to evolve and refine during residency training, with psychiatry trainees displaying a shift from avoidance to problem-focused approaches over time. Residents were also more likely to report less attachment insecurity after one year of training (Longitudinal Mixed Models - Figure 2).

Interpersonal competencies, sustained by mentalization skills, were observed as following a coupled longitudinal trajectory with burnout, so that an increase in interpersonal competencies was associated with a lower elevation in burnout dimensions (Figure 3).

**Image 1:**

**Figure:**
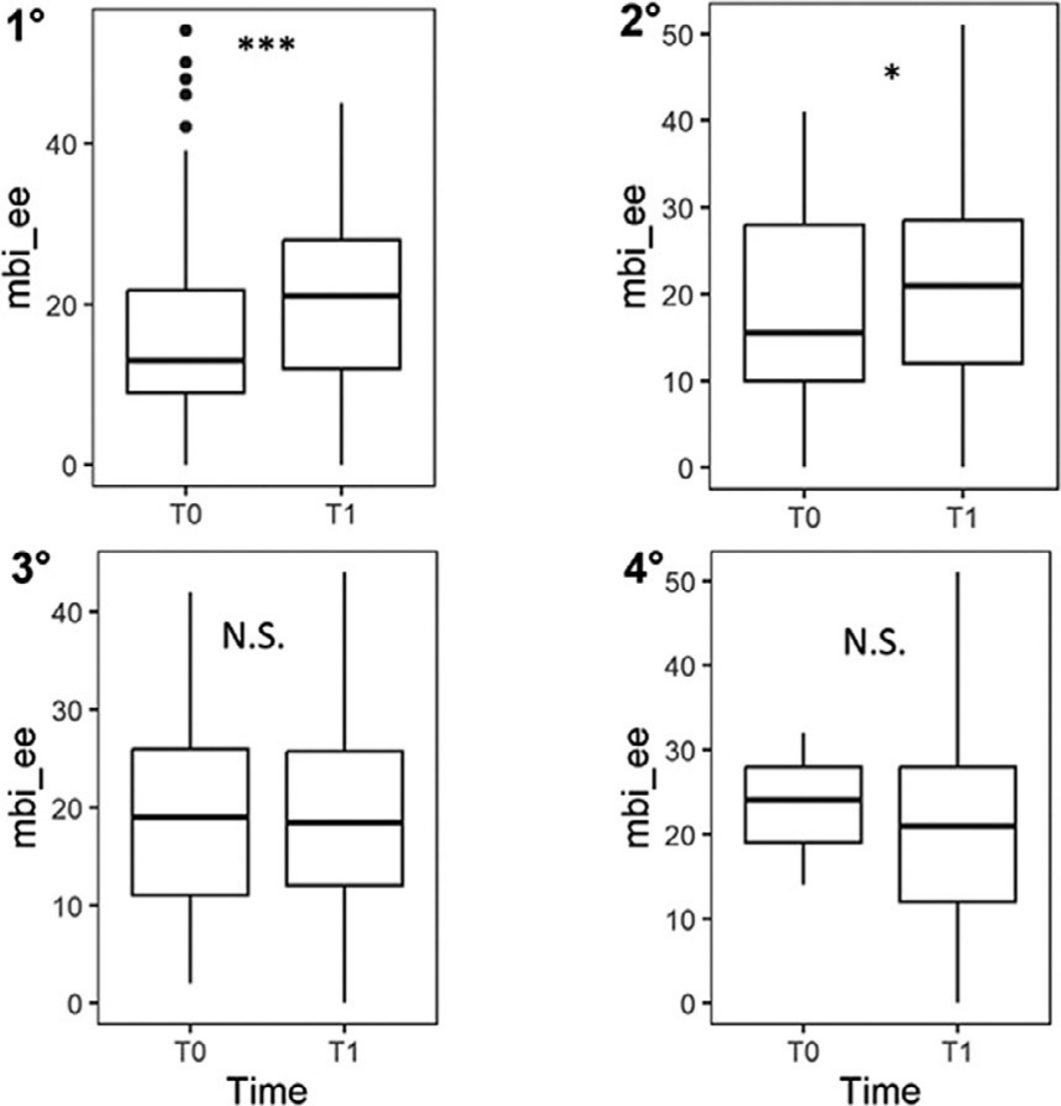

**Image 2:**

**Figure:**
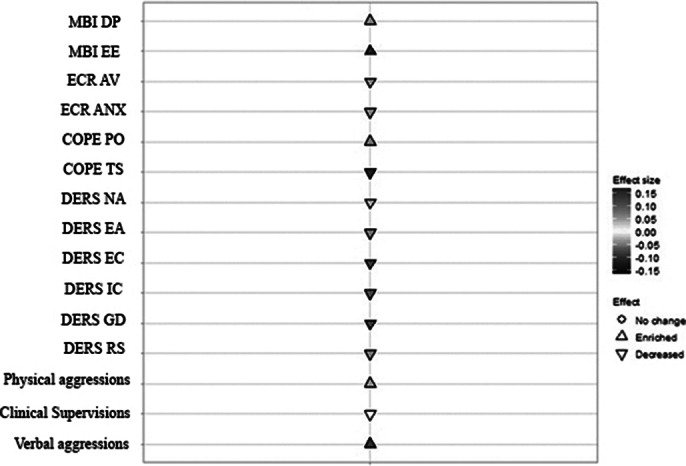

**Image 3:**

**Figure:**
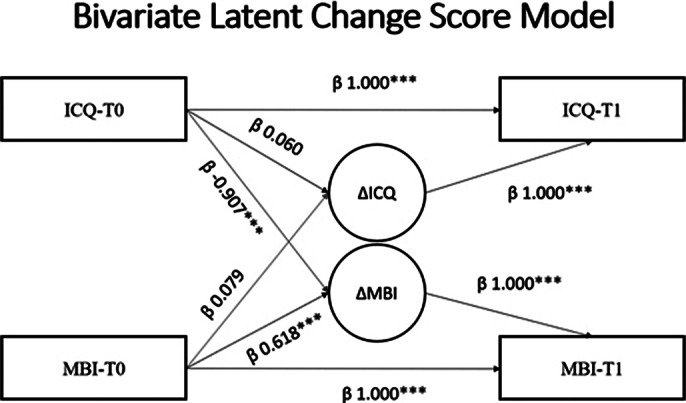

**Conclusions:**

Our findings suggest that enhancing emotional personal and interpersonal competencies could mitigate burnout, improve training outcomes, and potentially increase later workforce retention among psychiatry trainees. These results underscore the need for interventions targeting emotional competencies and mentalization during psychiatric training, such as focused clinical supervisions by senior staff members. Such interventions could enhance residents’ resilience, promote effective coping mechanisms, and ultimately improve the quality of psychiatric care.

**Disclosure of Interest:**

None Declared

